# The pan‐genome of the cultivated soybean (PanSoy) reveals an extraordinarily conserved gene content

**DOI:** 10.1111/pbi.13600

**Published:** 2021-06-15

**Authors:** Davoud Torkamaneh, Marc‐André Lemay, François Belzile

**Affiliations:** ^1^ Département de phytologie Faculté des sciences de l'agriculture et de l'alimentation (FSAA) Université Laval Québec Québec Canada; ^2^ Institut de biologie intégrative et des systèmes (IBIS) Université Laval Québec Québec Canada; ^3^ Department of Plant Agriculture University of Guelph Guelph Ontario Canada

**Keywords:** pan‐genome, soybean, *de novo* assembly, GmHapMap, genic PAV, long‐read sequencing

## Abstract

Studies on structural variation in plants have revealed the inadequacy of a single reference genome for an entire species and suggest that it is necessary to build a species‐representative genome called a pan‐genome to better capture the extent of both structural and nucleotide variation. Here, we present a pan‐genome of cultivated soybean (*Glycine max*), termed PanSoy, constructed using the *de novo* genome assembly of 204 phylogenetically and geographically representative improved accessions selected from the larger GmHapMap collection. PanSoy uncovers 108 Mb (˜11%) of novel nonreference sequences encompassing 3621 protein‐coding genes (including 1659 novel genes) absent from the soybean ‘Williams 82’ reference genome. Nonetheless, the core genome represents an exceptionally large proportion of the genome, with >90.6% of genes being shared by >99% of the accessions. A majority of PAVs encompassing genes could be confirmed with long‐read sequencing on a subset of accessions. The PanSoy is a major step towards capturing the extent of genetic variation in cultivated soybean and provides a resource for soybean genomics research and breeding.

## Introduction

The availability of reference genomes for most crops has created a crucial foundation for identification of genetic variants and subsequent genotype‐phenotype association studies. Until recently, all genetic variation in plants was identified based on the comparison of individual genomes to a single reference genome (Bayer *et al.,* 2020). Although this approach has allowed the efficient discovery of single‐nucleotide polymorphisms (SNPs) and large presence and absence variants (PAVs), DNA sequences that were not found in, or were highly diverged from, the reference genome are overlooked (Tao *et al.,* 2019a,b). One of the key features of structural variants (SVs; e.g. PAVs) is that they encompass large numbers of nucleotides and can consequently functionally impact a larger portion of the genome as compared to SNPs (Danilevicz *et al.,* 2020). Thus, this can result in substantial variation in the functional gene complement between individuals of the same species. The phenotypic relevance of SVs is becoming increasingly evident in plant genomes (Tao *et al.,* 2019a,b; Tranchant‐Dubreuil *et al.,* 2019).

To capture the variation in gene content among the individuals of the same species, the concept of the ‘pan‐genome’ was proposed (Tettelin *et al*., 2005). Broadly, a pan‐genome refers to the full complement of genes of a species and it is commonly partitioned into a set of core genes that are shared by all or most individuals and a set of variable genes that are partially shared (Tettelin *et al.,* 2005; Golicz *et al.,* 2016a,b). The variable genome represents diversity within the species; thus, its size is inherently linked with other genetic properties of the species, such as genome size, ploidy level, mode of reproduction, and bottlenecks during domestication. (Bayer *et al.,* 2020; Khan *et al.,* 2020; Tao *et al.,* 2019a,b). In general, three main approaches have been used to assemble a pan‐genome: comparative *de novo* assembly (Zhao *et al.,* 2018), iterative assembly (Hurgobin *et al.,* 2018), and the ‘map‐to‐pan’ strategy (Hu *et al.,* 2017; Wang *et al.,* 2018).

A growing number of pan‐genomes have been reported in the literature and these often encompass both the domesticated species and their wild progenitors (e.g. rice (*Oryza rufipogon* vs *Oryza sativa*), barley (*Hordeum spontaneum* vs *Hordeum vulgare*), wheat (*Triticum araraticum*, *Triticum turgidum* ssp. *dicoccoides* and *Triticum dicoccoides* vs *Triticum aestivum*), soybean (*Glycine soja* vs *G. max*) (Gao *et al.,* 2019; Golicz *et al.,* 2016a,b; Gordon *et al.,* 2017; Hirsch *et al.,* 2014; Hurgobin *et al.,* 2018; Li *et al.,* 2014a,b; Lin *et al.,* 2014; Liu *et al.,* 2020; Montenegro *et al.,* 2017; Wang *et al.,* 2018). Integrating crop wild relatives to assemble a genus‐level pan‐genome can help uncover genetic diversity that has been lost during domestication and breeding (Khan *et al.,* 2020). However, this can result in a larger pan‐genome with a higher proportion of variable genes than typically exist in the cultivated germplasm that forms the basis for a majority of plant breeding work.

Soybean, *G. max* (L.) Merr, is an ancient polyploid (paleopolyploid) and multiple evolutionary (two whole‐genome duplications) and co‐evolutionary forces (domestication and extensive selective breeding) have shaped its genome (Zhou *et al.,* 2015). Soybean is one of the first legume species with a complete genome sequence, and this has led to an unprecedented understanding of the genome organization and evolution in the Fabaceae (Schmutz *et al.,* 2010). Over the past few years, multiple new assemblies have been released for both cultivated [Zhonghuang 13 (Shen *et al.,* 2018) and Lee (Valliyodan *et al.,* 2019)] and wild (Li *et al.,* 2014a,b; Xie *et al.,* 2019) soybeans that have opened the door to understand soybean genome organization and to accelerate breeding.

The advent of high‐throughput sequencing technologies and availability of a high‐quality reference genome has provided an exceptional opportunity to systematically detect the DNA sequence variation among soybeans (Chung *et al.,* 2014; Fang *et al.,* 2017; Lam *et al.,* 2010; Maldonado dos Santos *et al.,* 2016; Song *et al.,* 2017; Torkamaneh *et al.,* 2017; Torkamaneh *et al.,* 2020; Valliyodan *et al.,* 2016; Zhou *et al.,* 2015). All these studies used a single reference genome (Williams 82 (Schmutz *et al.,* 2010)) to align reads and call variants. However, any DNA sequences that were absent from the reference genome were necessarily ignored. To overcome this shortcoming, two pan‐genomes were developed for soybean. An initial pan‐genome of *G. soja* was constructed by *de novo* assembly of seven accessions and this revealed that the *G. soja* pan‐genome is 30.2 Mbp larger than the genome of a single accession and ˜80% of the genes were present in all seven accessions (core) (Li *et al.,* 2014a,b). A much more exhaustive pan‐genome has been recently constructed by *de novo* assembly of 26 soybean accessions including 3 *G. soja* accessions, 9 *G. max* landraces, and 14 *G. max* cultivars. This broader work reached a very different conclusion, namely that ˜50% of genes in *G. soja* and *G. max* are dispensable (Liu *et al.,* 2020).

Here, in contrast, we present a pan‐genome focused uniquely and specifically on cultivated soybean (PanSoy). It is constructed using the *de novo* genome assembly of 204 phylogenetically and geographically representative accessions of improved *G. max* selected from the larger GmHapMap collection (Torkamaneh *et al.,* 2020). The objective of this study was to describe the entire gene repertoire in the cultivated soybean gene pool and thereby dispense with the added complexity of a non‐cultivated wild species.

## Results

### PanSoy assembly

**Figure 1:**
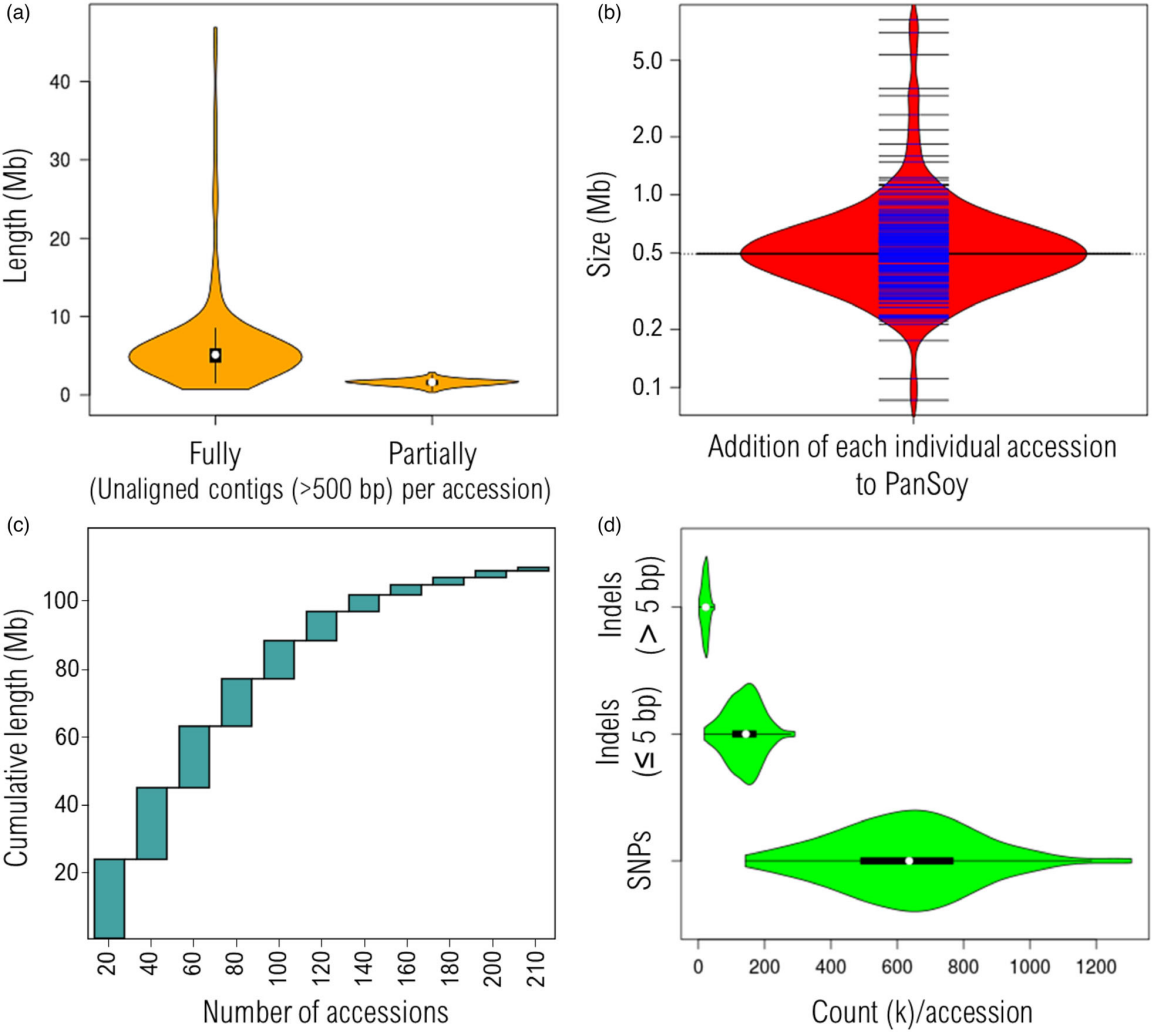
Composition of the PanSoy. (a) Distribution of fully and partially unaligned contigs among the 204 accessions of the soybean core set. (b) Contribution of each individual accession to the 108 Mb of novel sequences (average = ⁓680 kb; median = 504 kb). (c) Cumulative length of nonreference sequences based on incremental subsets (*n* = 20) of randomly selected accessions. Each bar represents the mean contribution of 100 random subsets. d Distribution of SNPs and indels (short ≤ 5 bp and long > 5 bp) among 204 soybean accessions of the core set compared with the Wm82 soybean reference genome.

On the basis of a cladogram of 1007 soybean accessions from the GmHapMap dataset (Torkamaneh *et al.,* 2020), 204 clusters encompassing the diversity among this large set of improved *G. max* accessions were defined to guide the construction of a *G. max* pan‐genome (PanSoy). In soybean, genome coverage plateaus (at ˜95%) at sequencing depths ≥15× (Figure S1a). Therefore, from each cluster, a single accession with sequencing depth ≥15× and mapping depth ≥10× was selected (Table S1, Figure S1b,c). *De novo* assembly of short reads was performed for each accession producing a total of 71 Gb of contigs ≥500 base pairs (bp) in size, for an average N50 value of 1.5 kb (Table S2). To obtain an estimate of the size of PanSoy, a sequence‐based pan‐genome using the ‘map‐to‐pan’ strategy (Hu *et al.,* 2017) was constructed by iteratively comparing each of the 204 sets of contigs to the soybean (cv. ‘Williams 82’) reference genome (Wm82.a4.v1) (Schmutz *et al.,* 2010) to identify previously unknown sequences. A total of 1 485 025 unaligned contigs (ranging from 500 to 32 kb) was obtained (Figure S2a). This totalled 1.9 Gb of novel sequences (1.6 and 0.3 Gb, respectively, of fully and partially unaligned sequences) with <90% identity to the reference genome, for an average of 7.6 Mb of fully unaligned sequence (contigs ≥ 500 bp) per accession (Figure 1a). After removing redundant and putative contaminating sequences (e.g. organellar or non‐soybean DNA), 160 066 unique novel contigs (ranging from 500 to 5 kb), totalling 108 Mb, were retained and collectively represent an addition of ⁓11% to the soybean reference genome (978 Mb). The average contribution of each individual accession was ⁓680 kb (median of 504 kb) (Figure 1b). Incremental random subsets of 20 accessions contributed progressively fewer megabases of novel sequence (Figure 1c). The final random subset of 24 accessions (the two last steps in the figure) contributed only 5 Mb to the total, suggesting that the core set used here had succeeded in capturing a vast majority of novel sequences not present in the Wm82 reference genome. We did not observe any correlation between the proportion of novel sequences per accession and the depth of coverage (Pearson’s *R^2^* = 0.005). On average, 629 K SNPs, 139 K short indels (≤5 bp), and 22 K long indels (>5 bp) per individual accession were detected relative to the Wm82 reference (Figure 1d). Finally, the extent of PanSoy sequence coverage was evaluated using a recently assembled reference genome ‘Lee’ (Valliyodan *et al.,* 2019), a genetically distant accession compared with Wm82. Almost all Lee genome sequences (99.91%) could be mapped to the PanSoy, whereas only 92% could be mapped to Wm82. Together, these results suggest that PanSoy offers an exhaustive characterization of the *G. max* genome.

A total of 3621 protein‐coding genes were predicted in the nonreference contigs. Of these, 1659 genes were identified as novel (<95% nucleotide identity to genes in the reference genome) and were expressed in one or more of the 101 tissues or conditions at ≥1 read per kilobase (kb) per million mapped reads (RPKM) in publicly available RNA‐sequencing (RNA‐Seq) data on 70 different *G. max* accessions (Table S3). In general, novel genes were shorter, owing to genes being predicted from relatively short contigs compared with the reference genome (Figure S2b). This could therefore lead to an underrepresentation of novel genes. PanSoy, including reference (Wm82; 978 Mb) and nonreference sequences (108 Mb), had a total size of 1086 Mb and 54 531 protein‐coding genes, 3% more than the reference Wm82 genome (52 872). On average, 93.2% of the 1440 single‐copy Embryophyta genes were completely assembled in the PanSoy based on the universal single‐copy orthologs (BUSCO) evaluation, showing high completeness of the gene annotation.

### PAV discovery and functional characterization

**Figure 2:**
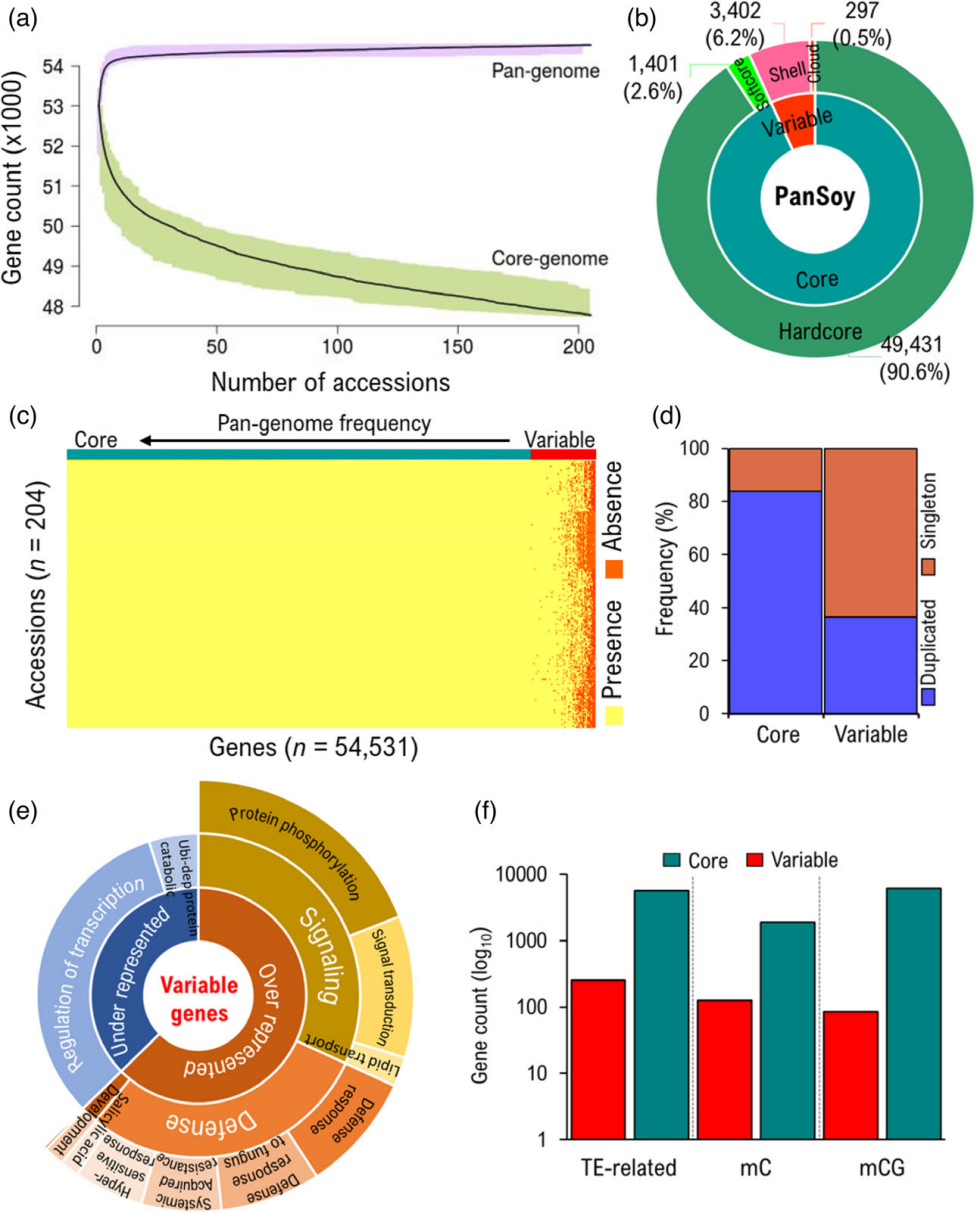
Genic composition of the PanSoy. (a) Capture of the pan‐ and core‐genome based on 100 randomized subsamples (with size of subsample varying from *n* = 1–204) of soybean accessions. The pan‐ and core‐genome curves were fitted using data points from all random subsamples and indicated by solid black lines. Upper and lower edges of the coloured areas (purple and green) correspond to the maximum and minimum numbers of genes present within a given subset of all accessions, respectively. (b) Composition of the PanSoy in terms of the subset of accessions within which a gene is found, thus defining the ‘core’ (≥95% of accessions) and ‘variable’ (<95%) sets of genes. (c) Landscape of genic PAVs. Genes are sorted by their occurrence with the highest frequency of occurrence on the left and the lowest on the right. (d) Frequency of duplicated vs. singleton genes in core vs. variable genome. e Functional classification of variable‐gene categories based on Gene Ontology (GO) term enrichment. f Proportion of TE‐related and methylated genes by gene category. mCG: genes methylated only in CG context; mC: genes methylated in all three contexts (CG/CHG/CHH).

A ‘map‐to‐pan’ strategy (Hu *et al.,* 2017) was used to determine gene presence or absence (PAV) after mapping all raw DNA sequences of the 204 accessions to the PanSoy. Then, genes with gene‐body coverage of ≥0.75 and CDS coverage ≥0.95 were considered as present in the genome. A total of 54 531 genes were detected among the 204 accessions, accounting for 100% of genes in the PanSoy. A relatively finite number of genes was observed in the PanSoy (Figure 2a). The extent of the pan‐genome size was estimated by iterative (*n* = 100) random sampling of accessions (*n* from 1 to 204). The resulting graph suggests a closed pan‐genome in which the vast majority of genic PAVs has been captured in this selected subset of accessions. According to the frequencies of presence of a gene among accessions used to build the PanSoy, we found 49 431 (90.6%) hardcore genes, that is present in >99% of the 204 accessions, as well as 1401 (2.6%) ‘softcore’ (95–99%), 3402 (6.2%) ‘shell’ (5‐95%) and 297 (0.5%) ‘cloud’ genes (<5%) (Figure 2b, Table S4). The most fascinating feature of PanSoy is its extremely high core‐gene content (>93%) (Figure 2c). As a result, each accession contains between 92.8% and 98.5% of these core genes and at most 7.2% of the variable (‘shell’ and ‘cloud’) genes.

Soybean has a highly duplicated genome (⁓75% of the genes present in multiple copies) as a result of two whole‐genome duplications (WGD) (Schmutz *et al.,* 2010). As described by Sankoff *et al*. (2010), paralog reduction (e.g. deletion of duplicated genes) followed WGD. Therefore, the expectation was to observe a higher proportion of duplicated genes in the variable genome. Contrary to this expectation, the PanSoy core genome was found to be enriched in duplicated genes compared with the variable portion of the genome (*P* < 0.01 (Tukey's HSD test)) (Figure 2d). However, a significantly higher (*P* < 1.8e−13, Welch two‐sample *t*‐test) ratio of non‐synonymous/synonymous (d*N*/d*S*) substitutions was observed in variable genes (d*N*/d*S* = 1.63) compared with core genes (d*N*/d*S* = 1.29), suggesting that variable genes are evolving faster with a lower functional constraint.

The core genome contained highly conserved genes that are more often annotated as ensuring essential functions (using Gene Ontology, GO) (e.g. regulation of transcription), whereas this category was significantly underrepresented (adjusted *P* value = 3.8e−3) in the variable genome. Compared with all genes captured in PanSoy, genes belonging to the variable genome were significantly enriched (adjusted *P* value < 0.01) in genes annotated as being involved in biological processes such as regulation of immune and defence responses, signalling, and plant development (Figure 2e, Table S5). Therefore, it could be hypothesized that, despite their relatively small number, these variable genes could still make important contributions to phenotypic variation for agronomic traits related to defence, signalling, and development. We also found that variable genes were clustered in certain genomic regions (e.g. 33–36 Mb on chromosome 16) (Figure S3) where resistance genes are significantly enriched (GO term enrichment; corrected *P* = 3.3e−31). Finally, we found that the core genome is enriched in both transposable element (TE)‐related and methylated genes (pairwise proportion test, *P* ≤ 1.7e−8 and *P* ≤ 2.6e−11, respectively) (Figure 2f) which might be attributed to the higher level of duplicated genes in the core genome.

**Figure 3:**
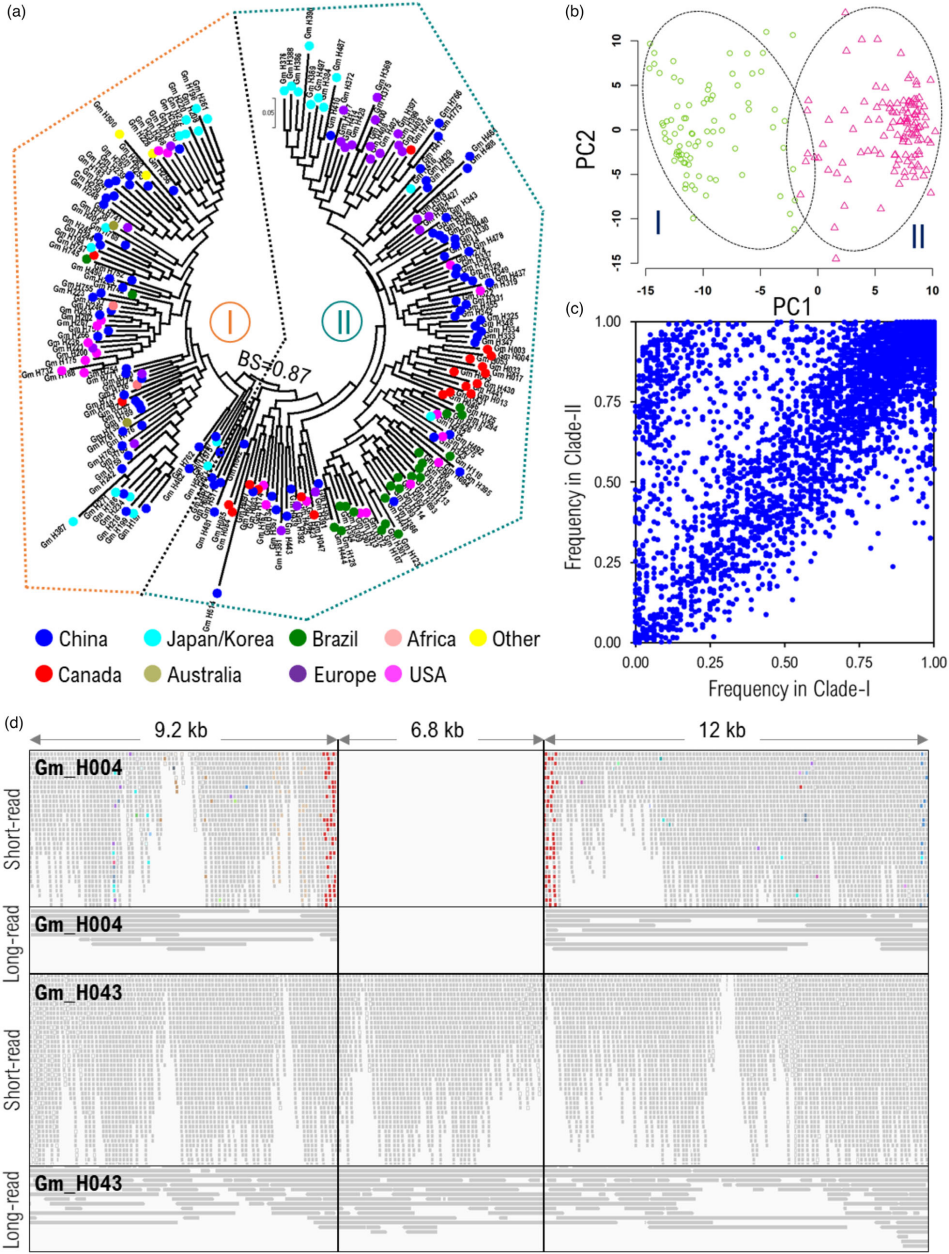
PanSoy population structure. (a) Maximum likelihood phylogenetic tree based on genic PAVs for 204 PanSoy accessions. The country of origin of accessions was differently coloured. (b) PCA plot of accession membership. (c) Scatter plot showing gene occurrence frequencies in Clade‐I and Clade‐II defined by the phylogenetic tree. (d) Short and long reads from Gm_H043 and Gm_H004 mapped to the Wm82 genome in a region of chromosome 3 where Gm_H004 contains a 6.8‐kb deletion that contains an annotated gene (Glyma.03G029300). The coloured dots in Gm_H004 short reads are mismatched nucleotides.

A maximum likelihood phylogenetic tree, principal component analysis, and model‐based (Bayesian) clustering based on PAVs split PanSoy accessions into two highly supported clades and clusters (Figure 3a,b, Figure S4). Although the PanSoy accessions are listed as having diverse geographical origins, no clear correlation was observed between geographical origin or maturity groups and phylogenetic topology. However, we noticed that almost all accessions from countries where soybean cultivation is relatively recent (e.g. Canada and Brazil) were grouped in clade II, while accessions from long‐established soybean‐growing countries (e.g. China, Korea, Japan and the US) were found in both clades. This grouping is largely consistent with a tree constructed using SNPs. A significant (*P* < 0.01) correlation was observed between distance matrices constructed using either PAVs or SNPs (Mantel test = 0.49) (Figure S4). Furthermore, the second clade, on average, contained a significantly (*P* < 3.1e−5, Welch two‐sample *t*‐test) lower gene loss (1436) than the first clade (1729) and possessed higher gene content (Figure 3c) that could be at least partially attributed to the intense selection and introgression of genes and alleles for disease resistance and abiotic stress tolerance from ancestral accessions into modern elite cultivars, especially when establishing the crop in novel areas (e.g. tropical soybean in Brazil, very early maturity in Canada).

### Long‐read sequencing and validation

Finally, to validate the PAVs that were called here based on the assembly of short reads, two accessions (Gm_H043 and Gm_H004) were selected for long‐read sequencing, thus enabling the direct detection of PAVs. This sequencing yielded 16.3 and 14.5 Gb of data, representing 17× and 15× depth of coverage and a read N50 of 7 and 22 kb for Gm_H043 and Gm_H004, respectively (Figure S5). After trimming, reads were aligned against the Wm82 reference genome and a comprehensive catalogue of structural variants, including deletions and insertions, was produced (Tables S6 and S7). To estimate the sensitivity and the precision of the PAVs, at first, the intervals spanned by deletions were compared with genic intervals to identify partial or complete overlaps (Figure 3d). This analysis revealed that 94.3% of the genic PAVs called on the basis of short reads were supported by the long‐read data. Secondly, on a broader level, unaligned short reads totalling 4.7 and 5.1 Mb of gDNA sequences from Gm_H043 and Gm_H004, respectively, were successfully mapped against the sequences of insertions identified via long reads. On average, 99% of the previously unaligned sequences could be mapped in this way, indicating the high quality of identification of novel nonreference sequences.

## Discussion

Despite the fact that the soybean reference genome (Wm82) is among the most complete plant reference genomes, it does not capture fully the genetic variability of this species (Liu *et al.,* 2020; Valliyodan *et al.,* 2019) leading to interest in producing a pan‐genome for this important crop. In doing so, it is important to define how broadly one wants to sample diversity. In many species, researchers have often chosen to capture the broader diversity within both in a crop species together with its immediate ancestral species (Gao *et al.,* 2019; Golicz *et al.,* 2016a,b; Gordon *et al.,* 2017; Hurgobin *et al.,* 2018; Liu *et al.,* 2020; Montenegro *et al.,* 2017; Wang *et al.,* 2018). Because such extended pan‐genomes typically capture a much larger number of variants than are present in the cultivated species alone, these can overestimate the diversity exploited by most breeders. In contrast, a pan‐genome consisting of cultivated accessions from a single species, such as PanSoy, aims to more closely reflect the diversity in cultivated soybean.

In soybean, both the wild progenitor (*G. soja*) and the cultivated species (*G. max*) belong to the same genus (Hymowitz and Newell, 1981). Recently, Liu *et al*. (2020) have developed what is likely the most exhaustive extended plant pan‐genome to date through re‐sequencing of close to 3000 accessions and *de novo* assembly of a representative subset of 26 selected wild, landrace, and cultivated soybean accessions based on a rich assortment of data (deep coverage with both short and long reads, optical mapping and Hi‐C data). In this recent work, more than twice the number of SNPs were identified compared with the number previously documented in *G. max* alone (31.9 vs ˜15 M SNPs; Fang *et al.,* 2017; Torkamaneh *et al.,* 2020; Zhou *et al.,* 2015). Importantly, these authors reported that only 50% of soybean genes were present in a large majority (>90%) of the 26 accessions, suggesting that the other half is quite variable in its presence among accessions, and close to 20% of genes in individual cultivated accessions were labelled as dispensable. This is in stark contrast to the results obtained in this work, where >94% of genes are shared by >90% of *G. max* accessions. Based on the publicly available data from the study of Liu *et al*. (2020), who do not distinguish between *G. soja* and *G. max* accessions, it is difficult to directly compare these two contrasting assessments of the degree of consistency in gene content among *G. max* accessions. Nonetheless, based on their definition of ‘dispensable genes’ (present in a minimum of 2 and maximum of 24 accessions), it is conceivable that a large set of these ‘dispensable genes’ are labelled as such due to variability in their presence within the three *G. soja* accessions. Any gene shared among all *G. max* accessions but absent from at least one *G. soja* accession would be labelled ‘dispensable’. The relative richness of the three *G. soja* accessions for different forms of genetic polymorphism was amply documented in this study. Wild soybeans were found to be significantly enriched (3.3×) in private (i.e. sample‐specific) structural variants compared with cultivated soybeans (average of 22.2% vs. 6.7%, respectively) (Liu *et al.,* 2020). Similarly, in examining the file summarizing SNP and indel variation, we find that over 50% of the indels observed within the 26 accessions used to construct the pan‐genome were found only within the subset of *G. soja* accessions. These data from Liu *et al*. (2020) suggest that the small set of three *G. soja* accessions were potentially the source of a disproportionate amount of the variation found and, conversely, that there is likely a greater amount of overlap in the gene sets among *G. max* accessions.

The primary goal of the present study was to accurately estimate the size of the core‐ and pan‐genome in a set of representative cultivated *G. max* accessions. Constructing a pan‐genome including wild relatives of the cultivated crops, commonly different species under the same genus, includes a sizeable genetic diversity that has been lost during domestication and selective breeding. These pan‐genomes that capture the complete gene repertoire at the genus level are called super pan‐genomes (Khan *et al.,* 2020). They undoubtedly provide important insights into evolutionary aspects of the organization the plants’ genome but do not necessarily reflect the diversity used by breeding programmes, as these mostly rely on elite accessions. Wild relatives present challenges in cultivar development as it is difficult to harness desirable genes by genetic recombination, and moreover, the concomitant introgression of undesirable genes from the wild parent can result in an inferior phenotype (Shakiba and Eizenga, 2014). Therefore, investigating genetic variability within the elite germplasm has broader applicability and higher interest for plant breeders.

The exceptionally high level of core‐gene content found in PanSoy, compared with other plant pan‐genomes (e.g. *Brassica oleracea* (81%) (Golicz *et al.,* 2016a,b); *Solanum lycopersicum* L. (74.2%) (Gao *et al.,* 2019); *Arabidopsis thaliana* (70%) (Contreras‐Moreira *et al.,* 2017); *T. aestivum* (64%) (Montenegro *et al.,* 2017); *Brassica napus* (62%) (Hurgobin *et al.,* 2018); *O. sativa* L. (54%) (Wang *et al.,* 2018)), is a striking feature but might be due the fact that PanSoy was constructed from improved accessions of *G. max* alone, while the recent pan‐genome of soybean (Liu *et al.,* 2020) and many other plants has often been constructed using mixed collections of wild, landrace and elite accessions (Gordon *et al.,* 2017; Tao *et al.,* 2019a,b), the former likely sharing fewer core genes with elite accessions. Interestingly, in tomato, a comparable species (e.g. genome size, rate of nucleotide variation, ploidy level, and outcrossing rate), the pan‐genome formed by domesticated elite accessions contained ˜90% of core genes (Gao *et al.,* 2019), a result quite similar to our findings. In another major diploid crop species, rice, a lower core‐gene content (˜83%) was observed among cultivated elite accessions (Wang *et al.,* 2018). This difference might be explained, however, by the fact that rice is divided into highly diverged groups of accessions (e.g. Xian/Indica and Geng/Japonica), reflecting a very different history of domestication and adaptation to broad geographic areas. In contrast, in many important soybean‐growing areas, modern‐day cultivars are known to have been derived from a relatively small number of founder lines (Hyten *et al.,* 2006). Similar to the wild and cultivated soybean pan‐genome (Liu *et al.,* 2020), a closed pan‐genome and an open core‐genome were observed in this study. This means that the pan‐genome size has reached a plateau, but that the core‐genome continues to decrease with every new genome included.

Gene Ontology (GO) enrichment analyses showed that the variable genes in PanSoy were significantly enriched in genes annotated as being involved in the regulation of immune and defence responses, signalling, and plant development. This is entirely consistent with previous findings in soybean (Li *et al.,* 2014a,b; Liu *et al.,* 2020) and other crops (Gao *et al.,* 2019; Gordon *et al.,* 2017; Wang *et al.,* 2018). The enrichment of genes involved in specific pathways or processes, such as defence and signalling, and their high evolutionary rates are consistent with a scenario in which variable genes evolve rapidly and are more likely to play important roles in phenotypic variation of agronomic traits in cultivated soybeans.

Similar to whole‐genome sequencing analysis, the exhaustiveness of PAV discovery in pan‐genome analysis is largely affected by the joint effect of sample size and sequencing depth. Early pan‐genome studies were based on deep sequencing (˜100×) and *de novo* assembly of relatively small numbers of individuals, 3–50 (Gan *et al.,* 2011; Golicz *et al.,* 2016a,b; Gordon *et al.,* 2017; Li *et al.,* 2014a,b; Schatz *et al.,* 2014; Song *et al.,* 2020). In such cases, the accurate estimation of pan‐ and core‐genome size is challenging due to the small sample size and the effects of phylogenetic distribution and population structure (Gordon *et al.,* 2017). These challenges can be overcome by increasing sample size but, despite the astounding reductions in the cost of DNA sequencing over the past decade, it can nonetheless be costly when large numbers of samples need to be deeply sequenced. More recently, pan‐genome studies using large numbers of samples with a medium sequencing depth (10–20×) have shown the statistical power commensurate to that of deep sequencing (Gao *et al.,* 2019; Wang *et al.,* 2018). Again, similar to what has been observed in whole‐genome sequencing analysis, the lower detection power in individual accessions can be compensated by increasing sample size (Wang *et al.,* 2016). Powered by a large sample size (204), we find that the total length of unique nonreference sequence reached a relative plateau suggesting that diversity within cultivated soybean had been well captured. Furthermore, the PanSoy collection has been selected as a representative core set from the larger GmHapMap collection (Torkamaneh *et al.,* 2020) to maximize the sampled genetic diversity among improved *G. max*. Lastly, to examine the extensiveness of the PanSoy, we assessed the extent of PanSoy sequence coverage using a recently assembled reference genome.

A final key feature of this work is the quality of the PanSoy. We first assessed the quality of PanSoy with conducting an extensive cross‐validation of PAVs via read mapping. We then evaluated the accuracy of PAVs via direct comparison with structural variants obtained from the long‐read sequencing. Together, these provide strong evidence that the PanSoy is of high quality. In conclusion, PanSoy sheds new light on the intraspecific variation in *G. max* and provides a platform to extensively explore, evaluate and characterize genetic diversity and evolution of cultivated soybean via investigation of its entire genomic repertoire.

## Methods

### Selection of the GmHapMap core collection

To extract a representative high‐quality core set among the 1007 accessions of GmHapMap (Torkamaneh *et al.,* 2020), PLINK (Purcell *et al.,* 2007) and the full set of nucleotide variants (⁓15 M) were used. Using the *Clustering* method (*‐‐cluster*), the complete collection was divided into 204 clusters (*‐‐K 204*). A single accession from each cluster was selected on the basis of sequencing depth (in all cases ≥15×; Table S1). The accessions within this core collection originate from 24 countries and five continents (Table S1). The raw DNA sequences of each accession were trimmed by Trimmomatic (v.0.36) (Bolger *et al.,* 2014) with following parameters ‘ILLUMINACLIP:2:30:10 LEADING:20 TRAILING:20 SLIDINGWINDOW:4:20 MINLEN:36’ before being used for *de novo* genome assembly.

### De novo assembly

To select the best *k‐mer* value, the genomes of a subset of soybean accessions (*n* = 5) were assembled using SOAPdenovo2 (v.240) (Luo *et al.,* 2012) using different *k‐mer* values based on a linear model ‘*K* = 2 * int (0.38 * (sequencing depth) + 10) + 1’. Then the best *k‐mer* value (*K* = 33) was identified based on the N50 of the resulting genome assemblies. The *de novo* assembly of 204 genomes was performed in parallel on a Linux system with Slurm Workload Manager using following command line for SOAPdenovo2 *SOAPdenovo‐63mer all ‐s configFile* *(*with average insertion length of *avg_ins=350)* *‐o output ‐K 33 ‐R ‐F*.

### Construction of the PanSoy

The PanSoy was constructed using the EUPAN pipeline (Hu *et al.,* 2017). In brief, the quality of each genome assembly was evaluated with QUAST (v. 2.357) (Gurevich *et al.,* 2013) using the latest version of the Wm82 soybean reference genome (Wm82.a4.v1) (Schmutz *et al.,* 2010). Then, unaligned contigs of at least 500 bp were extracted from the QUAST output and merged. This includes fully unaligned sequences (contigs with no alignment to the reference sequence) and partially unaligned sequences (contigs with at least one alignment and at least one unaligned fragment longer than 450 bp). Redundant sequences (>95% identity) were removed using CD‐HIT (v. 4.6.163) (Li and Godzik, 2006) with *‐c 0.9 ‐T 16 ‐M 50000* command line and a BLASTN ‘all‐versus‐all’ alignment approach. The unique nonreference contigs were mapped using BLASTN to the NT database (NCBI, November 2019) with parameters *‐evalue 1e‐5 ‐best_hit_overhang 0.25 ‐perc_identity 0.5 ‐max_target_seqs 10*. Using NCBI taxa identifiers and based on *E*‐values, contigs with the best alignment to the organellar genomes (chloroplast and mitochondria), microorganisms (e.g. bacteria, fungi, etc.), and non‐Viridiplantae (e.g. human and animals) were removed as possible contaminants. Finally, the PanSoy sequence was constructed by combining sequences of the Wm82 reference genome and the novel non‐redundant sequences.

### Annotation of the PanSoy

The GTF‐formatted gene and transcript annotation of the Wm82.a4.v1 reference genome was downloaded from Phythozome (https://phytozome.jgi.doe.gov/; Goodstein *et al.,* 2012). A combination of three different approaches (*ab initio* predictions, expression evidence, and protein homologies) in MAKER (release 2.31.8) (Cantarel *et al.,* 2008) was used to predict protein‐coding genes from novel nonreference sequences. Briefly, both RepeatMasker (http://www.repeatmasker.org/species/hg.html) and RepeatRunner (Duan *et al.,* 2019) were used to mask the repeats. Then, SNAP (Johnson *et al.,* 2008) and AUGUSTUS (Nachtweide and Stanke, 2019) were used for *ab initio* prediction with default parameters. Soybean expressed sequence tags (ESTs) and proteins were downloaded from GenBank and NCBI (January 2020) and aligned against novel non‐redundant nonreference sequences with BLASTN and BLASTX, respectively. Exonerate (Slater and Birney, 2005) and EVidenceModeler (Haas *et al.,* 2008) were used to realign and combine *ab initio* predictions with RNA and protein evidence. Finally, to identify novel genes, redundant genes (≥95% similar to the genes in Wm82) were removed.

### Evaluation of the PanSoy

To assess the quality of the PanSoy sequence and annotation, BUSCO (Benchmarking Universal Single‐Copy Orthologues) (v.2.032) (Simao *et al.,* 2015) was used to evaluate the newly assembled soybean reference genome ‘Lee’ and the PanSoy sequences. Then, RNA‐sequencing data sets from 101 different experiments on 70 different *G. max* accessions were downloaded from the NCBI SRA database (Table S3). All raw reads were combined in a single FASTQ file and aligned to the novel nonreference sequences using STAR (v.2.5.3a) (Dobin *et al.,* 2013) with default parameters.

### Determination of core and variable genes

Using a ‘map‐to‐pan’ strategy (Hu *et al.,* 2017), presence and absence variants (PAVs) among genic regions were identified by alignment of all raw DNA sequences of 204 accessions to the PanSoy using BWA (Li and Durbin, 2009). Then, gene‐body coverage and CDS coverage were estimated using BEDtools (v. 2.17.075) (Quinlan, 2014). Genes with CDS coverage of ≥0.95 and gene‐body coverage of ≥0.75 were considered present. The frequency of PAVs was estimated and genes were classified into two major categories: ‘core’ (loss rate < 0.05) and ‘variable’ genes (loss rate ≥ 0.05), and four subcategories: ‘hardcore’, ‘softcore’, ‘shell’, and ‘cloud’ genes, that is present in >99%, >95–99%, 5‐95%, and <5% of the 204 accessions, respectively. The heatmap distribution of core and variable genes was plotted with the heatmap function in R. To estimate the size of the pan‐genome and core‐genome, accessions from PanSoy were randomly sampled (*n* = 1–204) with 100 iterations and plotted with ggplot2 package in R (R Core Team, 2019).

### Functional analysis

The impact of variants was determined using SnpEff (Cingolani *et al.,* 2012). The analysis of GO terms was performed using all PanSoy genes with the GO Term Enrichment Tool integrated in SoyBase (Grant *et al.,* 2010) using Wm82 as the background. The list of duplicated genes, TE‐related genes, and methylated genes (mC and mCG) was extracted from Xu *et al*. (2018).

### Population structure

The population structure analysis was performed using PAVs. Principal component analysis and kinship were computed in TASSEL (v.5.1) (Glaubitz *et al.,* 2014) and visualized in R (R Core Team, 2019). Model‐based clustering of the 204 accessions was computed using a variational Bayesian inference implemented in fastSTRUCTURE (v1.0) (Raj *et al.,* 2014). Five runs were performed for each number of populations (*K*) from 1 to 10. Then, the final matrix of admixture proportions (*Q* matrix) of the best‐fit *K* value (*K* = 2) was used to visualize population structure and admixture. Maximum Likelihood (ML) phylogenetic analysis was performed in MEGA (v.7) (Kumar *et al.,* 2016) with bootstrap test (1000 replicates) to cluster taxa. Finally, the branches of the tree were aligned with the admixture classification.

### Long‐read sequencing using Oxford Nanopore MinION

Two soybean accessions (Gm_H043 and Gm_H004) were selected for long‐read sequencing using the Oxford Nanopore MinION technology. The DNA extraction for each accession was performed using different experimental procedures. (i) The DNA of Gm_H043 was extracted from trifoliate leaves harvested 3 weeks after germination and growth in a greenhouse. Leaves were flash‐frozen in liquid nitrogen upon harvest and stored in a −80°C freezer until DNA extraction. DNA was extracted using the Qiagen Gentra Puregene kit following grinding of leaves frozen in liquid nitrogen in a mortar and pestle. The extracted DNA was used for Oxford Nanopore library preparation without further processing. (ii) The DNA of Gm_H004 was extracted from trifoliate leaves harvested 2 weeks after germination and growth in the lab. Leaves were flash‐frozen in liquid nitrogen upon harvest and stored in a −80°C freezer until DNA extraction. DNA was extracted using a CTAB‐based protocol following grinding of the leaves using a Qiagen TissueLyser instrument. The CTAB protocol consisted of a 45‐min incubation in a CTAB lysis buffer to which RNase A was added, a cleaning step using a 24:1 chloroform:isoamyl alcohol mix, a precipitation using isopropanol, and two 70% ethanol washing steps. Approximately 9.5 µg of the extracted DNA were size‐selected on a BluePippin High‐Pass Plus cassette using a 15‐kb threshold. The DNA recovered in two fractions (original fraction + 0.1% Tween 20 fraction) was purified using SparQ PureMag beads. Finally, a genomic DNA library for each accession was constructed using the SQK‐LSK109 library preparation kit following the manufacturer's instructions. A total of ˜250 ng (Gm_H043) and ˜120 ng (Gm_H004) of the constructed library were loaded each on a single flowcell and run in a MinION.

### Bioinformatic processing of Oxford Nanopore data

Base calling was performed on the raw FAST5 data using Guppy (v. 3.2.4) (Wick *et al.,* 2019) on a GPU. Adapters were trimmed using Porechop (v. 0.2.4) (https://github.com/rrwick/Porechop). Reads were aligned against the Wm82 reference genome (including genomic, chloroplastic, and mitochondrial genomes) using NGMLR (v. 0.2.7) (Sedlazeck *et al.,* 2018) with default settings. Calling of structural variation was performed with Sniffles (v. 1.0.11) (Sedlazeck *et al.,* 2018) using default settings except for the minimum number of supporting reads and minimum length of reads which were set to 3 and 1 kb, respectively. Finally, an indexed FASTA file was created by the sequences of the insertions identified via long reads. Then, unaligned short reads from Gm_H043 and Gm_H004 were mapped against this FASTA file using BWA (Li *et al.,* 2009). The number of mapped and unmapped reads was calculated using SAMtools (Li *et al.,* 2009) from BAM files.

## Conflicts of interest

The authors declare that they have no competing interests.

## Author contributions

DT and FB conceived the project. DT carried out the development of PanSoy. MAL performed Oxford Nanopore long‐read sequencing and SV calling. DT and FB contributed to writing the manuscript.

## Supporting information


**Figure S1** Composition of the GmHapMap core collection.
**Figure S2** Assembly of PanSoy.
**Figure S3** Number of variable genes within 1‐Mb sliding windows across the soybean genome.
**Figure S4** Population structure analysis of PanSoy.
**Figure S5** Oxford Nanopore sequencing of Gm_H043 (left) and Gm_H004 (right).

**Table S1** List of PanSoy accessions.
**Table S2** Details of de novo assembly of PanSoy accessions.
**Table S3** List of RNA‐seq data sets downloaded from NCBI and used in this study.
**Table S4** List of gene PAVs (1 = present & 0 = absent) in PanSoy.
**Table S5** Description of GO analysis results of variable genes.
**Table S6** List of structural variants identified in Gm_H043 using Oxford Nanopre MinION sequencing data.
**Table S7** List of structural variants identified in Gm_H004 using Oxford Nanopre MinION sequencing data.

## Data Availability

The PanSoy data sets including PanSoy sequences and annotation, novel sequences, and a matrix of genic PAVs are publicly available at SoyBase (https://soybase.org/projects/SoyBase.C2021.01.php). The PanSoy data have also been added to the SoyBase Blast engine search (https://soybase.org/sequenceserver/).

## References

[pbi13600-bib-0001] Bayer, P.E. , Golicz, A.A. , Scheben, A. , Batley, J. and Edwards, D. (2020) Plant pan‐genomes are the new reference. Nat. Plants 6, 914–920. 10.1038/s41477-020-0733-0 32690893

[pbi13600-bib-0002] Bolger, A.M. , Lohse, M. and Usadel, B. (2014) Trimmomatic: a flexible trimmer for Illumina sequence data. Bioinformatics 30, 2114–2120. 10.1093/bioinformatics/btu170 24695404 PMC4103590

[pbi13600-bib-0003] Cantarel, B.l. , Korf, I. , Robb, S.m. , Parra, G. , Ross, E. , Moore, B. , Holt, C. *et al*. (2008) MAKER: an easy‐to‐use annotation pipeline designed for emerging model organism genomes. Genome Res. 18, 188–196. 10.1101/gr.6743907 18025269 PMC2134774

[pbi13600-bib-0004] Chung, W.‐h. , Jeong, N. , Kim, J. , Lee, W.k. , Lee, Y.‐g. , Lee, S.‐h. , Yoon, W. *et al*. (2014) Population structure and domestication revealed by high‐depth resequencing of Korean cultivated and wild soybean genomes. DNA Res. 21(2), 153–167. 24271940 10.1093/dnares/dst047 PMC3989487

[pbi13600-bib-0005] Cingolani, P. , Platts, A. , Wang, L.L. , Coon, M. , Nguyen, T. , Wang, L. , Land, S.J. *et al*. (2012) A program for annotating and predicting the effects of single nucleotide polymorphisms, SnpEff: SNPs in the genome of *Drosophila melanogaster* strain w1118; iso‐2; iso‐3. Fly 6, 80–92. 22728672 10.4161/fly.19695 PMC3679285

[pbi13600-bib-0006] Contreras‐Moreira, B. , Cantalapiedra, C.P. , García‐Pereira, M.J. , Gordon, S.P. , Vogel, J.P. , Igartua, E. , Casas, A.M. *et al*. (2017) Analysis of plant pan‐genomes and transcriptomes with GET_HOMOLOGUES‐EST, a clustering solution for sequences of the same species. Front. Plant Sci. 8, 184. 28261241 10.3389/fpls.2017.00184 PMC5306281

[pbi13600-bib-0007] Danilevicz, M.F. , Tay Fernandez, C.G. , Marsh, J.I. , Bayer, P.E. and Edwards, D. (2020) Plant pangenomics: approaches, applications and advancements. Curr. Opin. Plant Biol. 54, 18–25. 10.1016/j.pbi.2019.12.005 31982844

[pbi13600-bib-0009] Dobin, A. , Davis, C.A. , Schlesinger, F. , Drenkow, J. , Zaleski, C. , Jha, S. , Batut, P. *et al*. (2013) STAR: ultrafast universal RNA‐seq aligner. Bioinformatics 29, 15–21. 23104886 10.1093/bioinformatics/bts635 PMC3530905

[pbi13600-bib-0010] Duan, Z. , Qiao, Y. , Lu, J. , Lu, H. , Zhang, W. , Yan, F. , Sun, C. *et al*. (2019) HUPAN: a pan‐genome analysis pipeline for human genomes. Genome Biol. 20(1), 149. 10.1186/s13059-019-1751-y 31366358 PMC6670167

[pbi13600-bib-0011] Fang, C. , Ma, Y. , Wu, S. , Liu, Z. , Wang, Z. , Yang, R. , Hu, G. *et al*. (2017) Genome‐wide association studies dissect the genetic networks underlying agronomical traits in soybean. Genome Biol. 18, 161. 28838319 10.1186/s13059-017-1289-9 PMC5571659

[pbi13600-bib-0012] Gan, X. , Stegle, O. , Behr, J. , Steffen, J.G. , Drewe, P. , Hildebrand, K.L. , Lyngsoe, R. *et al*. (2011) Multiple reference genomes and transcriptomes for *Arabidopsis thaliana* . Nature 477, 419–423. 10.1038/nature10414 21874022 PMC4856438

[pbi13600-bib-0013] Gao, L. , Gonda, I. , Sun, H. , Ma, Q. , Bao, K. , Tieman, D.M. , Burzynski‐Chang, E.A. *et al*. (2019) The tomato pan‐genome uncovers new genes and a rare allele regulating fruit flavor. Nat Genet. 51, 1044–1051. 10.1038/s41588-019-0410-2 31086351

[pbi13600-bib-0014] Glaubitz, J.C. , Casstevens, T.M. , Lu, F. , Harriman, J. , Elshire, R.J. , Sun, Q.i. and Buckler, E.S. (2014) TASSEL‐GBS: a high capacity genotyping by sequencing analysis pipeline. PLoS One 9, e90346. 10.1371/journal.pone.0090346 24587335 PMC3938676

[pbi13600-bib-0015] Golicz, A.A. , Batley, J. and Edwards, D. (2016a) Towards plant pangenomics. Plant Biotechnol J. 14, 1099–1105. 10.1111/pbi.12499 26593040 PMC11388911

[pbi13600-bib-0016] Golicz, A.A. , Bayer, P.E. , Barker, G.C. , Edger, P.P. , Kim, H. , Martinez, P.A. , Chan, C.K.K. *et al*. (2016b) The pangenome of an agronomically important crop plant *Brassica oleracea* . Nat. Commun. 7, 13390. 27834372 10.1038/ncomms13390 PMC5114598

[pbi13600-bib-0017] Goodstein, D.M. , Shu, S. , Howson, R. , Neupane, R. , Hayes, R.D. , Fazo, J. , Mitros, T. *et al*. (2012) Phytozome: a comparative platform for green plant genomics. Nucleic Acids Res. 40, D1178–D1186. 22110026 10.1093/nar/gkr944 PMC3245001

[pbi13600-bib-0018] Gordon, S.P. , Contreras‐Moreira, B. , Woods, D.P. , Des Marais, D.L. , Burgess, D. , Shu, S. , Stritt, C. *et al*. (2017) Extensive gene content variation in the *Brachypodium distachyon* pan‐genome correlates with population structure. Nat. Commun. 8, 2184. 10.1038/s41467-017-02292-8 29259172 PMC5736591

[pbi13600-bib-0019] Grant, D. , Nelson, R.T. , Cannon, S.B. and Shoemaker, R.C. (2010) SoyBase, the USDA‐ARS soybean genetics and genomics database. Nucl. Acids Res. 84, D843–D846. 10.1093/nar/gkp798 PMC2808871 20008513

[pbi13600-bib-0020] Gurevich, A. , Saveliev, V. , Vyahhi, N. and Tesler, G. (2013) QUAST: quality assessment tool for genome assemblies. Bioinformatics 29, 1072–1075. 10.1093/bioinformatics/btt086 23422339 PMC3624806

[pbi13600-bib-0021] Haas, B.J. , Salzberg, S.L. , Zhu, W. , Pertea, M. , Allen, J.E. , Orvis, J. , White, O. *et al*. (2008) Automated eukaryotic gene structure annotation using EVidenceModeler and the Program to Assemble Spliced Alignments. Genome Biol. 9, R7. 10.1186/gb-2008-9-1-r7 18190707 PMC2395244

[pbi13600-bib-0022] Hirsch, C.N. , Foerster, J.M. , Johnson, J.M. , Sekhon, R.S. , Muttoni, G. , Vaillancourt, B. , Penagaricano, F. , *et al*. (2014) Insights into the maize pangenome and pan‐transcriptome. Plant Cell Online 26, 121–135. 10.1105/tpc.113.119982 PMC3963563 24488960

[pbi13600-bib-0023] Hu, Z. , Sun, C. , Lu, K.‐C. , Chu, X. , Zhao, Y. , Lu, J. , Shi, J. *et al*. (2017) EUPAN enables pan‐genome studies of a large number of eukaryotic genomes. Bioinformatics 33, 2408–2409. 10.1093/bioinformatics/btx170 28369371

[pbi13600-bib-0024] Hurgobin, B. , Golicz, A.A. , Bayer, P.E. , Kenneth Chan, C.‐K. , Tirnaz, S. , Dolatabadian, A. , Schiessl, S.V. *et al*. (2018) Homoeologous exchange is a major cause of gene presence/absence variation in the amphidiploid *Brassica napus* . Plant Biotechnol. J. 16, 1265–1274. 29205771 10.1111/pbi.12867 PMC5999312

[pbi13600-bib-0025] Hymowitz, T. and Newell, C.A. (1981) Taxonomy of the genusGlycine, domestication and uses of soybeans. Econ. Bot. 35, 272–288.

[pbi13600-bib-0026] Hyten, D.L. , Song, Q. , Zhu, Y. , Choi, I.Y. , Nelson, R.L. , Costa, J.M. , Specht, J.E. *et al*. (2006) Impacts of genetic bottlenecks on soybean genome diversity. Proc. Natl. Acad. Sci. USA. 10.1073/pnas.0604379103 PMC1624862 17068128

[pbi13600-bib-0028] Johnson, A.D. , Handsaker, R.E. , Pulit, S.l. , Nizzari, M.M. , O'Donnell, C.J. and de Bakker, P.I.W. (2008) SNAP: a web‐based tool for identification and annotation of proxy SNPs using HapMap. Bioinformatics 24, 2938–2939. 10.1093/bioinformatics/btn564 18974171 PMC2720775

[pbi13600-bib-0029] Khan, A.W. , Garg, V. , Roorkiwal, M. , Golicz, A.A. , Edwards, D. and Varshney, R.K. (2020) Super‐pangenome by integrating the wild side of a species for accelerated crop improvement. Trends Plant Sci. 25, 148–158. 10.1016/j.tplants.2019.10.012 31787539 PMC6988109

[pbi13600-bib-0030] Kumar, S. , Stecher, G. and Tamura, K. (2016) MEGA7: molecular evolutionary genetics analysis version 7.0 for bigger datasets. Mol. Biol. Evol. 33, 1870–1874. 10.1093/molbev/msw054 27004904 PMC8210823

[pbi13600-bib-0031] Lam, H.‐M. , Xu, X. , Liu, X. , Chen, W. , Yang, G. , Wong, F.‐L. , Li, M.‐W. *et al*. (2010) Resequencing of 31 wild and cultivated soybean genomes identifies patterns of genetic diversity and selection. Nat. Genet. 42, 1053–1059. 10.1038/ng.715 21076406

[pbi13600-bib-0032] Li, H. and Durbin, R. (2009) Fast and accurate short read alignment with Burrows‐Wheeler transform. Bioinformatics 25, 1754–1760. 10.1093/bioinformatics/btp324 19451168 PMC2705234

[pbi13600-bib-0033] Li, H. , Handsaker, B. , Wysoker, A. , Fennell, T. , Ruan, J. , Homer, N. , Marth, G. *et al*. (2009) The Sequence Alignment/Map format and SAMtools. Bioinformatics 25, 2078–2079. 10.1093/bioinformatics/btp352 19505943 PMC2723002

[pbi13600-bib-0034] Li, W. and Godzik, A. (2006) Cd‐hit: a fast program for clustering and comparing large sets of protein or nucleotide sequences. Bioinformatics 22, 1658–1659. 16731699 10.1093/bioinformatics/btl158

[pbi13600-bib-0035] Li, Y.H. , Zhou, G. , Ma, J. *et al*. (2014a) De novo assembly of soybean wild relatives for pan‐genome analysis of diversity and agronomic traits. Nat. Biotechnol. 32, 1045–1052. 25218520 10.1038/nbt.2979

[pbi13600-bib-0036] Li, Y.‐H. , Zhou, G. , Ma, J. , Jiang, W. , Jin, L.‐G. , Zhang, Z. , Guo, Y. *et al*. (2014b) De novo assembly of soybean wild relatives for pan‐genome analysis of diversity and agronomic traits. Nat. Biotechnol. 32, 1045–1052. 10.1038/nbt.2979 25218520

[pbi13600-bib-0037] Lin, K. , Zhang, N. , Severing, E. , Nijveen, H. , Cheng, F. , Visser, R. , Wang, X. , *et al*. (2014) Beyond genomic variation – comparison and functional annotation of three Brassica rapa genomes: a turnip, a rapid cycling and a Chinese cabbage. BMC Genom. 15, 250. 10.1186/1471-2164-15-250 PMC4230417 24684742

[pbi13600-bib-0038] Liu, Y. , Du, H. , Li, P. , Shen, Y. , Peng, H. , Liu, S. , Zhou, G.‐A. *et al*. (2020) Pan‐genome of wild and cultivated soybeans. Cell 182, 162–176.e13. 10.1016/j.cell.2020.05.023 32553274

[pbi13600-bib-0039] Luo, R. , Liu, B. , Xie, Y. , Li, Z. , Huang, W. , Yuan, J. , He, G. *et al*. (2012) SOAPdenovo2: an empirically improved memory‐efficient short‐read de novo assembler. Gigascience 1, 18. 10.1186/2047-217X-1-18 23587118 PMC3626529

[pbi13600-bib-0040] Maldonado dos Santos, J.V. , Valliyodan, B. , Joshi, T. , Khan, S.M. , Liu, Y. , Wang, J. , Vuong, T.D. *et al*. (2016) Evaluation of genetic variation among Brazilian soybean cultivars through genome resequencing. BMC Genom. 17, 110. 10.1186/s12864-016-2431-x PMC4752768 26872939

[pbi13600-bib-0042] Montenegro, J.D. , Golicz, A.A. , Bayer, P.E. , Hurgobin, B. , Lee, H.T. , Kenneth Chan, C.‐K. , Visendi, P. *et al*. (2017) The pangenome of hexaploid bread wheat. Plant J. 90, 1007–1013. 28231383 10.1111/tpj.13515

[pbi13600-bib-0043] Nachtweide, S. and Stanke, M. (2019) Multi‐genome annotation with AUGUSTUS. Methods Mol. Biol. 1962, 139–160. 10.1007/978-1-4939-9173-0_8 31020558

[pbi13600-bib-0044] Purcell, S. , Neale, B. , Todd‐Brown, K. , Thomas, L. , Ferreira, M.A.R. , Bender, D. , Maller, J. *et al*. (2007) PLINK: a tool set for whole genome association and population‐based linkage analyses. Am. J. Hum. Genet. 81, 559–575. 17701901 10.1086/519795 PMC1950838

[pbi13600-bib-0045] Quinlan, A.R. (2014) BEDTools: the Swiss‐Army tool for genome feature analysis. Curr. Protoc. Bioinf. 47, 1–34. 10.1002/0471250953.bi1112s47 PMC4213956 25199790

[pbi13600-bib-0046] R Core Team . (2019) R: A Language and Environment for Statistical Computing. Vienna: R Foundation for Statistical Computing. http://www.r‐project.org/index.html

[pbi13600-bib-0047] Raj, A. , Stephens, M. and Pritchard, J.K. (2014) fastSTRUCTURE: variational inference of population structure in large SNP data sets. Genetics 197, 573–589. 24700103 10.1534/genetics.114.164350 PMC4063916

[pbi13600-bib-0048] Sankoff, D. , Zheng, C. and Zhu, Q. (2010) The collapse of gene complement following whole genome duplication. BMC Genom. 11, 313. 10.1186/1471-2164-11-313 PMC2896955 20482863

[pbi13600-bib-0049] Schatz, M.C. , Maron, L.G. , Stein, J.C. , Wences, A.H. , Gurtowski, J. , Biggers, E. , Lee, H. *et al*. (2014) Whole genome de novo assemblies of three divergent strains of rice, *Oryza sativa*, document novel gene space of aus and indica. Genome Biol. 15:506. 25468217 10.1186/s13059-014-0506-z PMC4268812

[pbi13600-bib-0050] Schmutz, J. , Cannon, S.B. , Schlueter, J. , Ma, J. , Mitros, T. , Nelson, W. , Hyten, D.L. *et al*. (2010) Genome sequence of the palaeopolyploid soybean. Nature 463, 178–183. 20075913 10.1038/nature08670

[pbi13600-bib-0051] Sedlazeck, F.J. , Rescheneder, P. , Smolka, M. , Fang, H. , Nattestad, M. , von Haeseler, A. and Schatz, M.C. (2018) Accurate detection of complex structural variations using single‐molecule sequencing. Nat Methods 15, 461–468. 10.1038/s41592-018-0001-7 29713083 PMC5990442

[pbi13600-bib-0052] Shakiba, E. and Eizenga, G.C. (2014). Unraveling the secrets of rice wild species. In: Yan, W. and Bao, J. , (eds.) Rice ‐ Germplasm, Genetics and Improvement. InTech, UK. 1–58. 10.5772/58393

[pbi13600-bib-0053] Shen, Y. , Liu, J. , Geng, H. , Zhang, J. , Liu, Y. , Zhang, H. , Xing, S. *et al*. (2018) novo assembly of a Chinese soybean genome. Sci. China Life Sci. 61, 871–884. 30062469 10.1007/s11427-018-9360-0

[pbi13600-bib-0054] Simão, F.A. , Waterhouse, R.M. , Ioannidis, P. , Kriventseva, E.V. and Zdobnov, E.M. (2015) BUSCO: assessing genome assembly and annotation completeness with single‐copy orthologs. Bioinformatics 31, 3210–3212. 26059717 10.1093/bioinformatics/btv351

[pbi13600-bib-0055] Slater, G.S. and Birney, E. (2005) Automated generation of heuristics for biological sequence comparison. BMC Bioinformatics 6, 31. 15713233 10.1186/1471-2105-6-31 PMC553969

[pbi13600-bib-0056] Song, J.‐M. , Guan, Z. , Hu, J. , Guo, C. , Yang, Z. , Wang, S. , Liu, D. *et al*. (2020) Eight high‐quality genomes reveal pan‐genome architecture and ecotype differentiation of *Brassica napus* . Nat. Plants 6, 34–45. 10.1038/s41477-019-0577-7 31932676 PMC6965005

[pbi13600-bib-0057] Song, Q. , Yan, L. , Quigley, C. , Jordan, B.D. , Fickus, E. , Schroeder, S. , Song, B.H. *et al*. (2017) Genetic characterization of the soybean nested association mapping population. Plant Genome 10(2), 10.3835/plantgenome2016.10.0109 28724064

[pbi13600-bib-0058] Tao, Y. , Jordan, D.R. and Mace, E.S. (2019a) Crop genomics goes beyond a single reference genome. Trends Plant Sci. 24, 1072–1074. 10.1016/j.tplants.2019.10.001 31648939

[pbi13600-bib-0059] Tao, Y. , Zhao, X. , Mace, E. , Henry, R. , Jordan, D. *et al*. (2019b) Exploring and exploiting pan‐genomics for crop improvement. Mol. Plant. 12, 156–169. 30594655 10.1016/j.molp.2018.12.016

[pbi13600-bib-0060] Tettelin, H. , Masignani, V. , Cieslewicz, M.J. , Donati, C. , Medini, D. , Ward, N.L. , *et al*. (2005) Genome analysis of multiple pathogenic isolates of Streptococcus agalactiae: implications for the microbial "pan‐genome". Proc Natl Acad Sci USA. 102(39), 13950–13955. 10.1073/pnas.0506758102. 16172379 PMC1216834

[pbi13600-bib-0061] Torkamaneh, D. , Laroche, J. , Tardivel, A. , O’Donoughue, L. , Cober, E. , Rajcan, I. and Belzile, F. (2017) Comprehensive description of genome‐wide nucleotide and structural variation in short‐season soybean. Plant Biotechnol. J. 16, 749–759. 28869792 10.1111/pbi.12825 PMC5814582

[pbi13600-bib-0062] Torkamaneh, D. , Laroche, J. , Valliyodan, B. , O'Donoughue, L. , Cober, E. , Rajcan, I. , Abdelnoor, R.V. *et al*. (2020) Soybean haplotype map (GmHapMap): a universal resource for soybean translational and functional genomics. Plant Biotechnol. J. 19, 324–334. 10.1111/pbi.13466 32794321 PMC7868971

[pbi13600-bib-0063] Tranchant‐Dubreuil, C. , Rouard, M. and Sabot, F. (2019) Plant pangenome: impacts on phenotypes and evolution. Ann. Plant Rev. 2(2), 10.1002/9781119312994.apr0664

[pbi13600-bib-0064] Valliyodan, B. , Cannon, S.B. , Bayer, P.E. , Shu, S. , Brown, A.V. , Ren, L. , Jenkins, J. *et al*. (2019) Construction and comparison of three reference‐quality genome assemblies for soybean. Plant J. 100, 1066–1082. 31433882 10.1111/tpj.14500

[pbi13600-bib-0065] Valliyodan, B. , Qiu, D. , Patil, G. , Zeng, P. , Huang, J. , Dai, L.U. , Chen, C. *et al*. (2016) Landscape of genomic diversity and trait discovery in soybean. Sci. Rep. 6, 23598. 27029319 10.1038/srep23598 PMC4814817

[pbi13600-bib-0066] Wang, H. , Xu, X. , Vieira, F. , Xiao, Y. , Li, Z. , Wang, J. , Nielsen, R. *et al*. (2016) The power of inbreeding: NGS‐based GWAS of rice reveals convergent evolution during rice domestication. Mol. Plant. 9, 975–985. 10.1016/j.molp.2016.04.018 27179918

[pbi13600-bib-0067] Wang, W. , Mauleon, R. , Hu, Z. , Chebotarov, D. , Tai, S. , Wu, Z. , Li, M. *et al*. (2018) Genomic variation in 3,010 diverse accessions of Asian cultivated rice. Nature 557, 43–49. 10.1038/s41586-018-0063-9 29695866 PMC6784863

[pbi13600-bib-0068] Wick, R.R. , Judd, L.M. and Holt, K.E. (2019) Performance of neural network basecalling tools for oxford nanopore sequencing. Genome Biol. 20, 129. 10.1186/s13059-019-1727-y 31234903 PMC6591954

[pbi13600-bib-0070] Xie, M. , Chung, C.‐L. , Li, M.‐W. , Wong, F.‐L. , Wang, X. , Liu, A. , Wang, Z. *et al*. (2019) A reference‐grade wild soybean genome. Nat. Commun. 10, 1216. 30872580 10.1038/s41467-019-09142-9 PMC6418295

[pbi13600-bib-0071] Xu, C. , Nadon, B.D. , Kim, K.D. and Jackson, S.A. *et al*. (2018) Genetic and epigenetic divergence of duplicate genes in two legume species. Plant Cell Environ. 2018, 1–12. 10.1111/pce.13127 29314059

[pbi13600-bib-0072] Zhao, Q. , Feng, Q. , Lu, H. , Li, Y. , Wang, A. , Tian, Q. , *et al*. (2018) Pan‐genome analysis highlights the extent of genomic variation in cultivated and wild rice. Nat Genet. 50(2), 278–284. 10.1038/s41588-018-0041-z. 29335547

[pbi13600-bib-0073] Zhou, Z. , Jiang, Y.u. , Wang, Z. , Gou, Z. , Lyu, J. , Li, W. , Yu, Y. *et al*. (2015) Resequencing 302 wild and cultivated accessions identifies genes related to domestication and improvement in soybean. Nat Biotechnol. 33, 408–414. 10.1038/nbt.3096 25643055

