# Supplementary material for: The pan‐genome of the cultivated soybean (PanSoy) reveals an extraordinarily conserved gene content
Source: Plant Biotechnol J. 2021 Jun 15;19(9):1852–62. doi: 10.1111/pbi.13600 (PMC8428833; doi:10.1111/pbi.13600)
Supplement: Supplementary file 1 — Figure S1 Composition of the GmHapMap core collection. Figure S2 Assembly of PanSoy. Figure S3 Number of variable genes within 1‐Mb sliding windows across the soybean genome. Figure S4 Population structure analysis of PanSoy. Figure S5 Oxford Nanopore sequencing of Gm_H043 (left) and Gm_H004 (right). [file PBI-19-1852-s002.docx]

# **The Pan-genome of the Cultivated Soybean (PanSoy) Reveals an Extraordinarily Conserved Gene Content**

Davoud Torkamaneh^1,2,3^, Marc-André Lemay^1,2^ and François Belzile^1,2*^

^1^ Département de phytologie, Faculté des sciences de l’agriculture et de l’alimentation (FSAA), Université Laval, Québec (Québec), Canada

^2^ Institut de biologie intégrative et des systèmes (IBIS), Université Laval, Québec (Québec), Canada

^3^ Department of Plant Agriculture, University of Guelph, Guelph (Ontario), Canada

*****Corresponding author: François Belzile ([francois.belzile@fsaa.ulaval.ca](mailto:francois.belzile@fsaa.ulaval.ca))

# **Supplementary Figures**


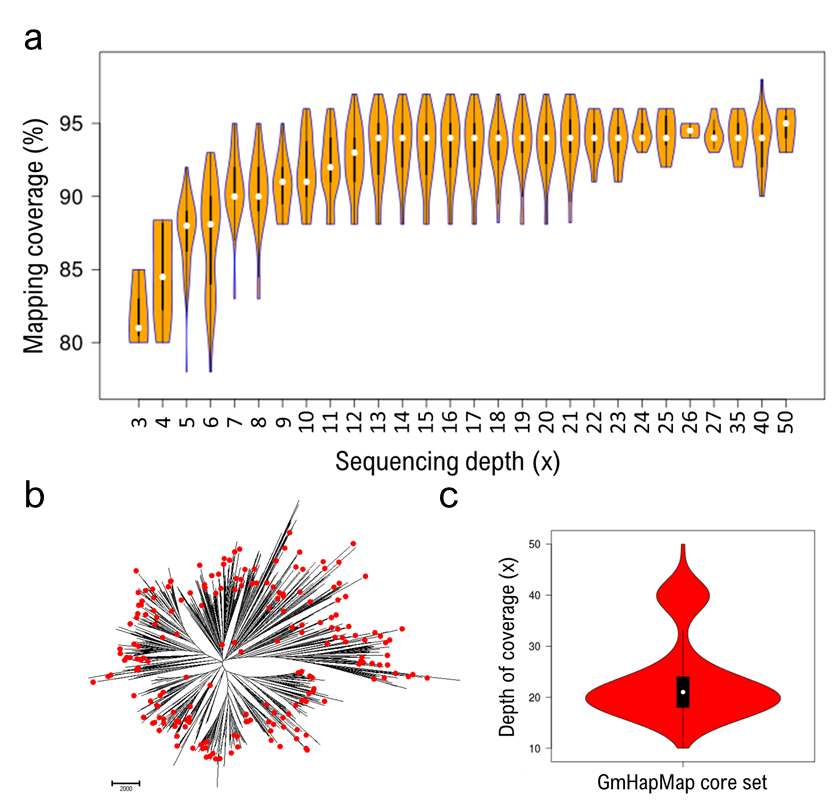


**Supplementary Figure 1. Composition of the GmHapMap core collection. a** Genome coverage of the 1,007 resequenced soybean accessions (GmHapMap), following alignment against the Wm82 reference genome, as a function of sequencing depth. **b** Cladogram of the 1,007 GmHapMap accessions based on 12M SNPs. Red dots indicate the accessions selected to be part of the core set used for producing the PanSoy genome. **c** Distribution of the depth of coverage for the subset of 204 core-set accessions.


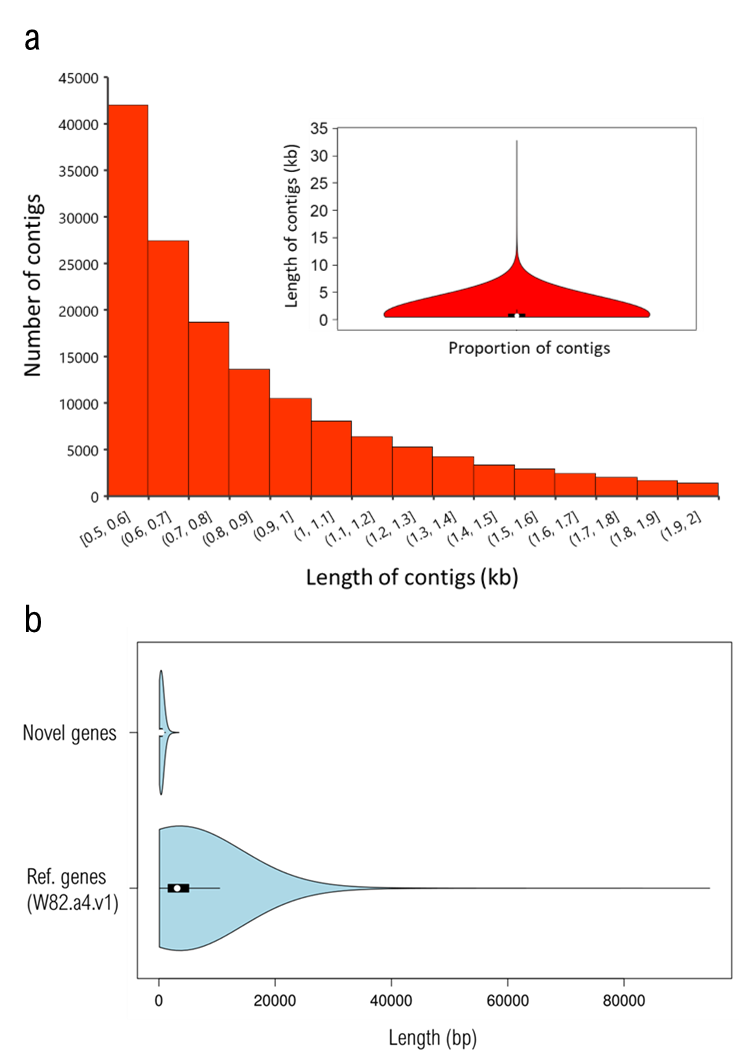


**Supplementary Figure 2. Assembly of PanSoy. a** Length distribution of unaligned contigs. **b** Length distribution of novel vs. Wm82 reference genome genes.

**Supplementary Figure 3.** Number of variable genes within 1-Mb sliding windows across the soybean genome.


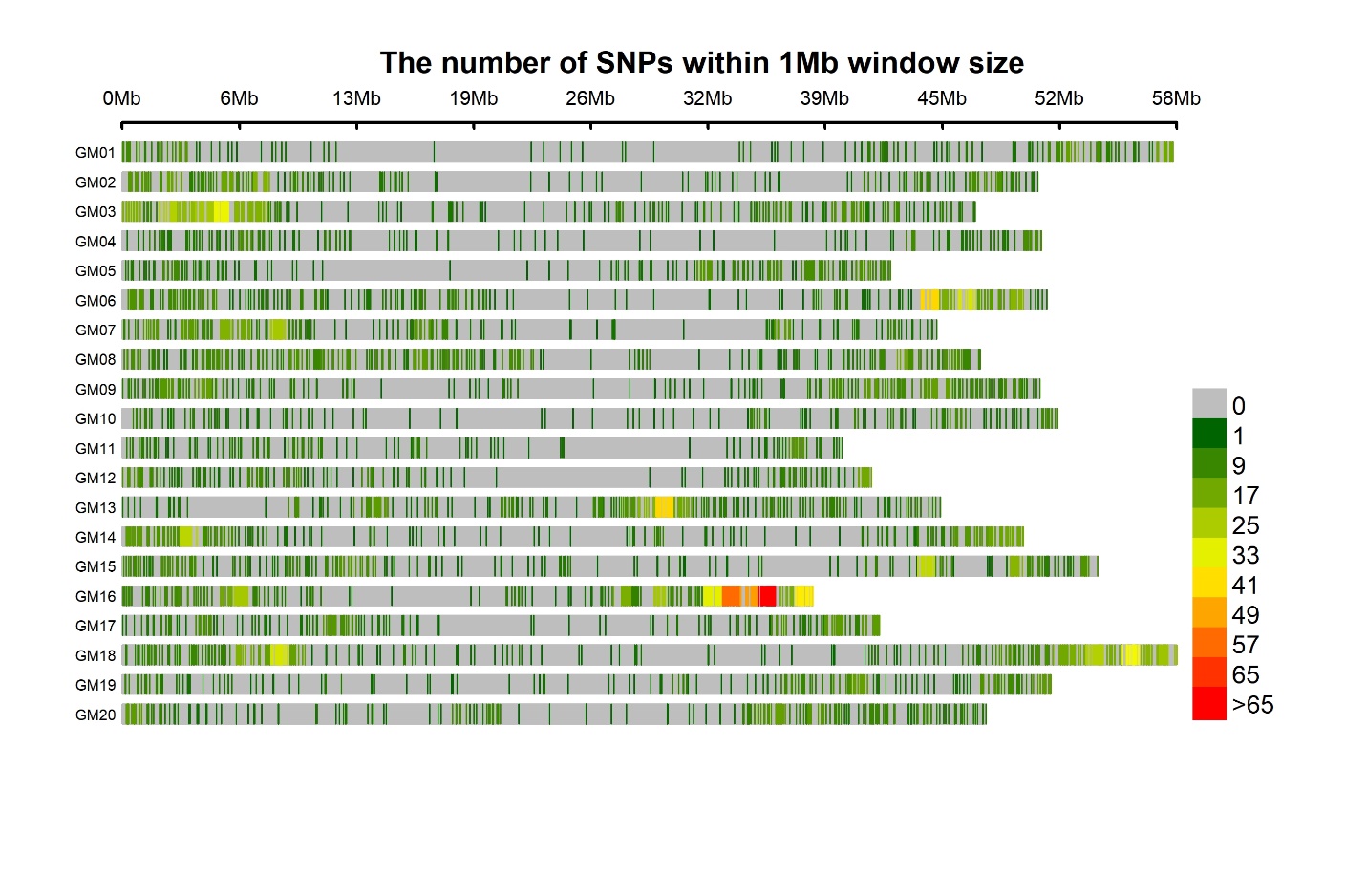


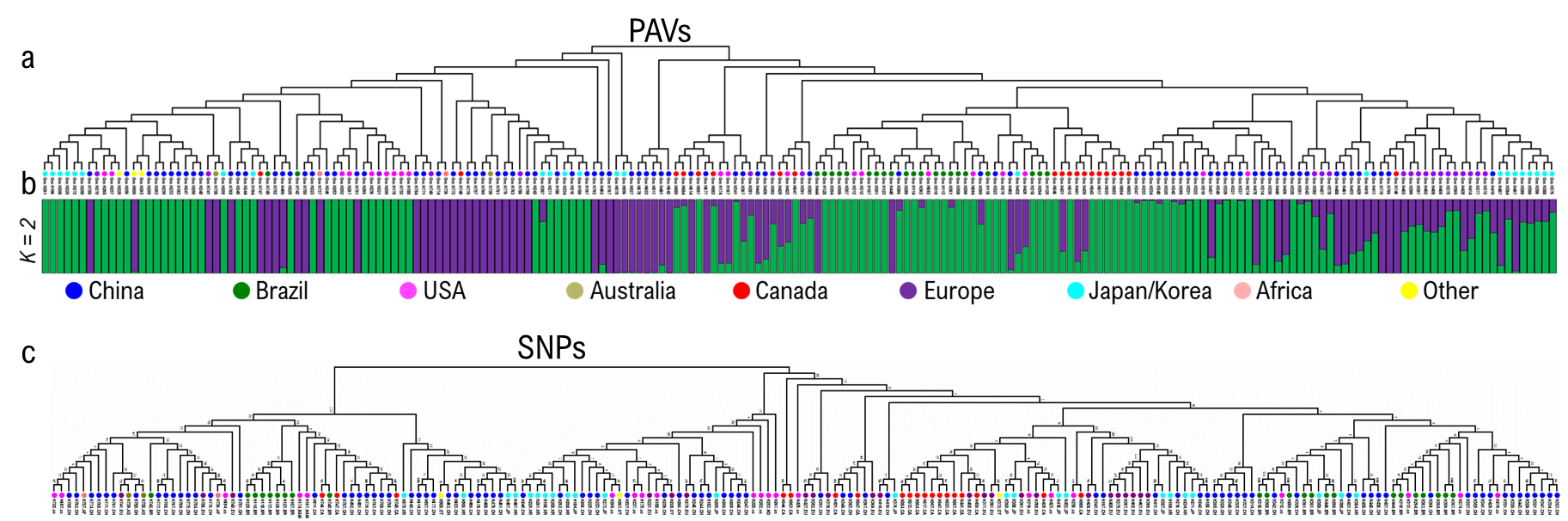


**Supplementary Figure 4. Population structure analysis of PanSoy. a** Maximum likelihood phylogenetic tree based on genic PAVs for 204 soybean accessions. **b** Bayesian clustering plot of individual membership based on genic PAVs to optimal K = 2 STRUCTURE groups. **c** Maximum likelihood phylogenetic tree based on SNPs for 204 soybean accessions.


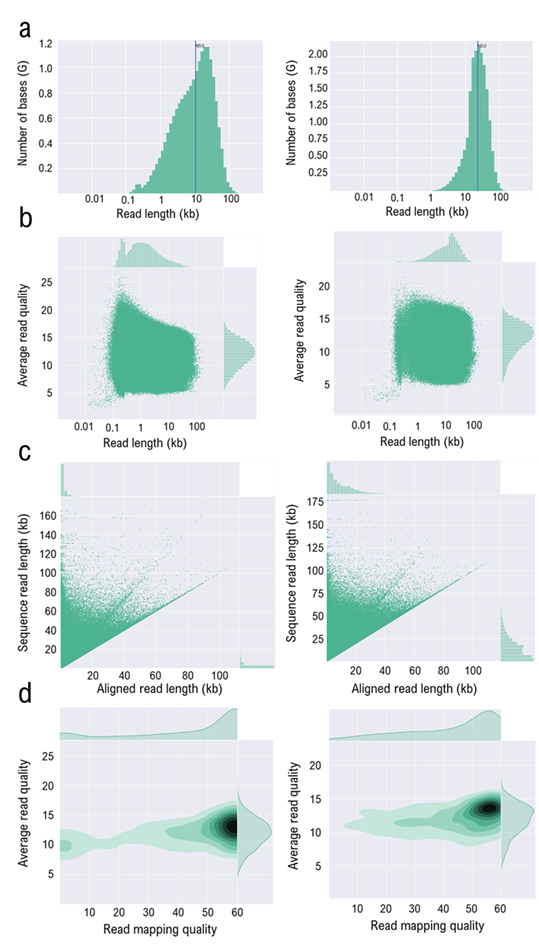


**Supplementary Figure 5. Oxford Nanopore sequencing of Gm_H043 (left) and Gm_H004 (right). a** Weighted histogram of read lengths after log transformation. **b** Read length vs. average read quality plot using dots after log transformation of read lengths. **c** Aligned read length vs. sequenced read length plot using dots. **d** Read mapping quality vs. average base-call quality plot using a kernel-density estimation. Plots were made using NanoPack (De Coster et al., 2018).
